# Auditory Cortex Responses to Clicks and Sensory Modulation Difficulties in Children with Autism Spectrum Disorders (ASD)

**DOI:** 10.1371/journal.pone.0039906

**Published:** 2012-06-29

**Authors:** Elena V. Orekhova, Marina M. Tsetlin, Anna V. Butorina, Svetlana I. Novikova, Vitaliy V. Gratchev, Pavel A. Sokolov, Mikael Elam, Tatiana A. Stroganova

**Affiliations:** 1 Institute of Neuroscience and Physiology, University of Gothenburg, Gothenburg, Sweden; 2 Moscow State University of Psychology and Education, Moscow, Russia; 3 Moscow Pediatric Center, Moscow, Russia; 4 Clinical Department for the Study of Adolescent Psychiatry, Mental Health Research Center of Russian, Academy of Medical Sciences, Moscow, Russia; Centre Hospitalier Le Vinatier (Bât. 452), France

## Abstract

Auditory sensory modulation difficulties are common in autism spectrum disorders (ASD) and may stem from a faulty arousal system that compromises the ability to regulate an optimal response. To study neurophysiological correlates of the sensory modulation difficulties, we recorded magnetic field responses to clicks in 14 ASD and 15 typically developing (TD) children. We further analyzed the P100m, which is the most prominent component of the auditory magnetic field response in children and may reflect preattentive arousal processes. The P100m was rightward lateralized in the TD, but not in the ASD children, who showed a tendency toward P100m reduction in the right hemisphere (RH). The atypical P100m lateralization in the ASD subjects was associated with greater severity of sensory abnormalities assessed by Short Sensory Profile, as well as with auditory hypersensitivity during the first two years of life. The absence of right-hemispheric predominance of the P100m and a tendency for its right-hemispheric reduction in the ASD children suggests disturbance of the RH ascending reticular brainstem pathways and/or their thalamic and cortical projections, which in turn may contribute to abnormal arousal and attention. The correlation of sensory abnormalities with atypical, more leftward, P100m lateralization suggests that reduced preattentive processing in the right hemisphere and/or its shift to the left hemisphere may contribute to abnormal sensory behavior in ASD.

## Introduction

Apart from the ‘core’ deficits central to a diagnosis of autism, such as abnormal social interaction, communication and presence of repetitive behaviors, individuals with autism spectrum disorders (ASD) frequently demonstrate a range of sensory abnormalities. Sensory difficulties are observed in both high- and low-functioning ASD individuals and are prominent from the first years of life [1], through childhood [2], [3], and during adulthood [4], [5]. They cover different sensory domains and may manifest as both hyper- and hyposensitivity to stimulation.

In many cases sensory abnormalities are especially noticeable in the auditory domain. Parents of ASD children may suspect hearing impairment or hearing loss in their children during the first two years of life, because of their striking unresponsiveness to sound [6]. On the other hand, hypersensitivity to sound or hyperacusis is also a very common problem in ASD and may even require therapeutic intervention [7]. Van England et al. [8] investigated electrodermal responses to auditory stimuli in children with autism and found that many of these children lacked autonomic responses to the first acoustic event in a series, but once responding they demonstrated high amplitude electrodermal responses. Ben-Sasson and colleagues [1] reported remarkably frequent co-occurrence of auditory hyper- and hypo-sensitivity symptoms in children with ASD and suggested that both of these problems may be explained by a common mechanism, such as a dysfunctional arousal system that compromises the ability to regulate an optimal response [1]. In spite of multiple evidence for the presence of auditory sensory modulation difficulties in ASD individuals, their neurofunctional correlates have not yet been investigated.

Electro- and Magnetoencephalography (EEG and MEG) methods have good time resolution, allowing investigation of auditory processing stages affected in autism. Using EEG we have recently found a reduction of the temporal N1c (also called Tb) component of the auditory event-related potential (ERP) in 4–8 year-old children with autism [9]. The N1c reduction was observed in response to auditory clicks presented after long silent intervals (first click in a pair) and was limited to the right hemisphere (RH). The typically developing children demonstrated predominantly rightward stimulus-locked EEG phase synchronization in the N1c time range, while the opposite leftward predominance was observed in children with autism. We suggested that the N1c group differences might reflect abnormalities in initial orienting/arousal in autism. The networks for alerting, attention-orienting and rapid detection of stimuli are normally rightward lateralized in the brain [10]–[12]. We proposed, therefore, that the decreased N1c response in the RH might reflect aberrant functioning of these circuits in children with autism. We did not investigate, however, whether these EEG findings were related to the presence of sensory abnormalities or severity of autism. Furthermore, the limited EEG electrode array in our previous study precluded source analysis of the observed effects.

In the present study we used MEG to investigate brain sources of these abnormal responses to clicks in ASD children, and look for correlation of these abnormalities with sensory processing difficulties.

The primary and secondary auditory areas at the superior and lateral temporal surfaces (core, belt, and parabelt) are the major sources of the obligatory auditory cortical responses [13], [14]. Although MEG is largely insensitive to radial sources at the lateral temporal regions generating N1c/Tb [15], it is sensitive to the tangential sources that make a major contribution to the N100m and P100m components of auditory magnetic field response in children [16]–[20].

The P100m component at around 100 ms after stimulation is the most prominent component of the auditory evoked magnetic field response in children, whereas the child N100m is of lower amplitude than in adults [16]. The P100m amplitude decreases and the N100m amplitude increases during child development [16], [21]. Similar age-related effects have been described using EEG. Specifically, amplitude of the positive P100 ERP component decreases and the amplitude of negative N100 (N1b) ERP component increases at midline electrodes between approximately 8 to 16 years of age [15], [22]. It has been proposed that these age dynamics reflect cortical maturation processes that take place on a layer-by-layer basis in the cerebral cortex. The large ‘positive’ P100(m) peak observed in younger children might represent recurrent activation of layers III and IV, while the generation of N100(m) might be dominated by activation of layers upper III and II [23]–[25]. Maturation of axons in layer IV and deeper part of layer III is already finished by 6th year of life, while maturation of cortico-cortical axons in the upper layers II and III occurs between 6–12 years [25], [26]. Thus, the P100(m) in school-age children may more closely reflect mature thalamic input to layers III–IV, while gradually increasing N100(m) to a greater extent reflects maturation of cortico-cortical connections. Considering different origin of the P100(m) and N100(m), their abnormalities may reflect different dysfunctions associated with neuro-developmental disorders.

In this study we focused on the P100m component of the magnetic field response. *First*, this component can be reliably identified in the majority of children because of its high amplitude [16]. *Second*, similarly to the P50 (P1) component in adults [27], [28] the child P100m may be modulated by the reticular activation system (RAS) and may reflect arousal regulation abnormalities and related sensory modulation difficulties in ASD.

To record auditory magnetic fields we used a ‘paired click’ paradigm similar to that used in our previous study [9]. We have previously suggested that processing of temporally novel auditory stimuli might be impaired in autism [9]. We expected, therefore, to find greater between-group differences in response to the first click in a pair, presented after a long silent interval.

To assess sensory abnormalities we used two psychometric instruments. *First*, we applied Short Sensory Profile (SSP) questionnaire [29], which has previously been shown to reliably separate between ASD children and typically developing or developmentally delayed children without autism [30]–[32]. The total SSP score was used in order to assess in children sensory modulation difficulties across different domains.

Putative auditory magnetic field abnormalities in ASD children might to a great extent reflect atypical auditory behavior, rather than general sensory modulation difficulties. Unlike e.g. tactile problems, the auditory processing difficulties in autism decrease with age [33] and are most evident in early life. Therefore, as a *second* step, we applied the questionnaire by Dahlgren and Gillberg (1989) that among other items contained questions concerning presence of auditory sensory modulation problems during the first two years of life. We speculated that children who had severe auditory modulation abnormalities during infancy and toddlerhood might have more disrupted magnetic field responses to clicks even if behavioral symptoms had diminished with age.

## Materials and Methods

### Participants

Fourteen children diagnosed with autism spectrum disorder (one girl) and fifteen age-matched typically developing control children (two girls) participated in the study. None of these children participated in our previous EEG study of auditory processing in autism [9]. One ASD child was ambidextrous and one control child was left-handed. The rest were right-handed according to the parent questionnaire that included eighteen questions about child's hand preference during everyday activities. All children were free from medication with neuro-active drugs for at least 1 month before the investigation. Their hearing was normal according to available medical records. IQ in all participants was assessed with Kaufman Assessment Battery for Children (KABC-II). The diagnosis of ASD (autism in 6 children, Asperger's disorder in 6 children, and Pervasive Developmental Disorder (PDD) – Not Otherwise Specified in 2 children) was based on DSM-IV-TR criteria and was made by an experienced clinician (V.V.G.). Parents of all children were also presented with Russian translation version of the Social Communication Questionnaire (SCQ-*Lifetime*, [34]. In addition, parents of all ASD and 14 of 15 typically developing children filled in the Autism spectrum Quotient (AQ) for children [35]. All but one child (S#7) in the ASD group scored above SCQ cut-off for pervasive developmental disorders. All but one child (S#9) in the ASD group also scored above AQ cut-off for the ASD. Taking into account approximately 95% sensitivity of AQ and SCQ questionnaires for diagnosis of ASD/PDD, such result would be expected to happen by chance. Therefore, subjects who scored below AQ/SCQ cut-offs have not been excluded. All typically developing children scored below AQ and SCQ cut-offs. Information on participant's age and IQ, AQ and SCQ scores is summarized in table 1.

**Table 1:** Demographic information.

	ASD mean (SD), *N = 14*	Control mean (SD), *N = 15*
Age (months)	127 (27)	128 (19)
Sequential IQ	91 (19)	111 (12)
Simultaneous IQ	98 (17)	122 (14)
General IQ	92 (18)	120 (14)
Child AQ	87 (26)	60 (10)*
SCQ-*Lifetime*	24.2 (6)	7.1 (2.8)

* AQ was available in 14 of 15 control subjects.

Presence of sensory abnormalities in all subjects was tested using Russian translation of the SSP. The parents also filled in the questionnaire concerning autism-related symptoms during the first two years of life [6] and assessed severity of each symptom on a 10-point scale. Among 130 questions this questionnaire contained six questions about presence of auditory sensory modulation difficulties.

The study was approved by the local ethics committee of the Moscow University of Psychology and Education and was conducted following the ethical principles regarding human experimentation (Helsinki Declaration). A written informed consent was obtained from a parent/guardian of each child.

### Experimental paradigm

Stimuli were 4 ms white noise clicks presented in pairs (S1, S2), with 1000 ms intervals within the pair (S1 to S2) and randomly varying 8–11 sec intervals between the pairs (S1 to S1). The stimuli were presented with equal probability to the right ear (R), left ear (L), or binaurally (B). The side of presentation (B, R or L) was always the same within a S1–S2 pair. In total, 102 pairs of clicks of each type were presented during six blocks, each lasting for approximately 7 minutes. Stimuli were delivered via plastic ear tubes inserted in the ear channels. The tubes were fixated to the MEG helmet in order to minimize possible noise resulting from their contact with the subject's clothing.

In 9 children the sounds were presented at 80 dB over the hearing threshold, defined separately for left and right ears using monaural clicks. In remaining children, we used the SPL level corresponding to 80 dB over the hearing threshold previously defined for 20 healthy adults (95 dB SPL for both ears). The mean SPL level did not differ between ASD and typically developing children. During the experiment, participants watched a silent video of their choice and were instructed to ignore the auditory stimulation.

### MEG recording and pre-processing

MEG was recorded in a sitting position in a neuromagnetically-shielded room using a 306-channel MEG (Vectorview, Elekta-Neuromag) comprising 204 orthogonal planar gradiometers and 102 magnetometers in 102 locations above the participant's head. The temporal signal space separation (tSSS) and movement compensation (movecomp) options implemented by MaxFilter (Elekta-Neuromag) were used to suppress interference signals generated outside the brain, as well as to compensate for head movements. The data were converted to standard head position (x = *0* *mm; y = 0* *mm; z = 45* *mm*).

The magnetic fields were recorded at 1000 Hz and were filtered off-line with a bandpass of 1–100 Hz. Signal periods of 2500 ms were extracted such as to include 500 ms before and 2000 ms after the S1 stimuli. The epochs were excluded if signal amplitude exceeded 2000 fT/cm for gradiometers or 12,000 fT for magnetometers in either direction. For the rest of the data, biological artefacts (cardiac fields, eye movements, myogenic activity) were corrected using independent component analysis (ICA) implemented by EEGlab software [36], separately for gradiometers and magnetometers. Prior to ICA, the data dimensionality was reduced to 64 principal components. The timecourses and spatial distributions of the ICs were visually inspected and the components describing artefacts were rejected. This typically resulted in rejection of 2–5 components for each sensor type. The artefact corrected data were filtered with a 40 Hz low-pass filter. For each stimulus type the epochs comprising −350 to 500 ms relative to stimulus onset were extracted. The average number of artefact free epochs per condition was 94.2 (68–102) in the ASD group and 97.6 (76–102) in the control group and did not significantly differ between the groups (p>0.25 for all conditions).

### Structural MRI

High-resolution structural T1-weighted MRIs were acquired on a 1.5T Toshiba ExcelArt Vantage scanner (TR = 12 ms, TE = 5 ms, flip angle  = 20°, 160 sagittal slices, slice thickness  = 1.0 mm, voxel size  = 1.0×1.0×1.0 mm^3^). A representation of the cortical surface was constructed from the individual structural MRIs with the FreeSurfer software [37], [38]. Cortical white matter was segmented in the high resolution MRIs, and the estimated border between gray and white matter was tessellated, providing a triangular representation of the surface. The surface was also ‘inflated’ to unfold cortical sulci, providing a convenient viewing of cortical activation patterns [38].

### Magnetoencephalography source analysis

Cortical sources of the MEG signals were estimated using the distributed model, the weighted minimum norm estimate [39] implemented by MNE software (http://www.nmr.mgh.harvard.edu/martinos). For each participant the boundary element model and forward model were calculated based on individual T1-weighted MRI using the FreeSurfer software package. For each hemisphere the model contained 4098 dipole elements (sources) that overlaid realistic representation of the cortical surface. The inverse operator was constructed with depth weighting [40], using ‘weightexp’ parameter of 0.7 and ‘weightlimit’ parameter of 8. To allow flexibility of the model against small co-registration errors, orientations of the dipole elements were not strictly constrained to be perpendicular to the cortical surface, and a “loose orientation constraint parameter” of 0.3 was used [41]. In addition to the MNE, the noise-normalized MNE, called dynamic statistical parametric map (dSPM), was also calculated [42]. The dSPM converts the MNE into a statistical test variable that is essentially the signal-to noise ratio of the current estimate at each spatial location. Thus, dSPM identifies locations where the MNE amplitudes are above the noise level. To facilitate comparison between subjects, the individual MNE and dSPM cortical distributions were morphed to the ‘fsaverage’ template brain provided by FreeSurfer.

### Regions of interest (ROIs) definition

**Figure 1:**
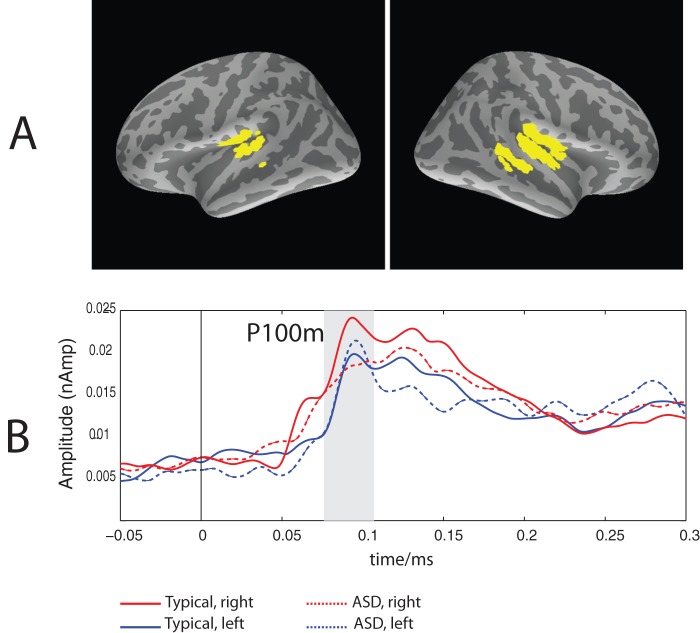
The P100m ROIs (A) and the grand average MNE current time courses in these ROIs (B) for the 1^st^ binaural click in a pair. Gray bar denotes the P100m time window.

To define the ROIs we used data-driven approach based on activation overlap between the subjects. For each cortical source we calculated the number of subjects demonstrating its significant activation (dSPM, p<0.05) in response to at least one type of stimuli (S1 or S2) at some time point during 76–108 ms interval. Although in a few cases the P100m peaked after 108 ms, this time limit was used in order to decrease possible contribution of N100m activity in the P100m ROI definition. The liberal p<0.05 threshold emphasized maximal overlap between subjects, rather than favoring a few subjects displaying greatest response amplitudes. The left and right ROI in the vicinity of the auditory cortex were created based on activation overlap between the subjects. The vertex source was included into the ROI if activation at this vertex was observed in more than 50% of either ASD or control subjects. The left and right ROIs are shown in figure 1(A).

### Assessment of P100m parameters in the ROIs

Some children demonstrated prominent N100m component that followed P100m and was also located in the vicinity of the auditory cortex. Direction of current in superior temporal surface (‘outgoing’ for P100m and ‘ingoing’ for N100m) clearly differentiated between these components (see Material S1). To ensure that the peak activity in the P100m ROI describes P100m rather than N100m activation, we applied the following approach. *First*, at the left and right superior temporal surfaces we defined regions characterized by outgoing ‘positive’ direction of current in the P100m time range (see Material S2). *Second*, the time course of the mean signed MNE values was calculated in these regions and the latency of the maximal *positive* peak (P100m) in the 76–130 ms time range was found. This latency was considered to be the P100m peak latency. In some subjects no P100m peak was observed in either right (in two ASD subjects) or left (in two control subjects) hemisphere. Therefore, we further analyzed the mean P100m amplitude in the 76–108 ms range.

### Statistical analysis

In this study we analyzed only binaural responses due to their higher amplitude and better signal to noise ratio. Repeated measures ANOVAs with factors Group (ASD vs. control), Stimulus Order (S1, S2) and Hemisphere were performed for the P100m components' amplitude and latency parameters. Spearman rank order correlations are reported through the paper. Application of other statistical test is described in the Result section. The false discovery rate (FDR) correction for multiple testing under dependency [43] has been applied, when appropriate.

## Results

### Sensory abnormalities

The mean SSP scores for ASD and typical control children are summarized in table 2. The ASD children scored lower on the SSP-*total*, as well as on all its sections, except the Movement Sensitivity. According to the formal cut-off for the total SSP score, none of the 14 ASD children performed in the normal range, 3 children had ‘probable sensory differences’ and 11 had ‘definite differences’. In the control group three children had ‘definite differences’ and two children – ‘probable differences’. The rest performed within the normal range.

**Table 2:** Short Sensory Profile results.

Section	ASD mean (SD), N = 14	Typical mean (SD), N = 15	*F*
*Tactile Sensitivity*	27.7 (4.6)	31.4 (2.0)	7.9*
*Taste*	12.1 (5.5)	16.2 (3.3)	6.0*
*Movement Sensitivity*	12.6 (2.1)	13.4 (3.0)	0.6, ns
*Underresponsive/Seeks Sensation*	21.2 (4.9)	28.6 (4.5)	17.8**
*Auditory Filtering*	18.4 (5.6)	23.0 (4.7)	5.7*
*Low Energy/Weak*	16.8 (5.6)	25.7 (3.6)	26.5***
*Visual/Auditory Sensitivity*	18.1 (4.6)	22.1 (1.9)	8.5**
*SSP-total*	126.9 (20.6)	160.4 (15.1)	25.1***

* p<0.05, **p<0.01, ***p<0.001, FDR corrected.

Table 3 shows differences between ASD and typical children in auditory behavior during early life. The total score composed of all these items most reliably differentiated between the groups. Five of the ASD children had the total scores (30 to 58) exceeding the maximal score in the typical sample (24). Analysis of separate items listed in table 3 has shown that difference between these five children and the rest of the ASD group was due to their higher sensitivity to sound. They were more likely then the other ASD children to *react strongly to sound regardless of level* (Q3) (Mann-Whitney U Test, Z = 2.9, p<0.01, uncorrected), *put fingers in the ears* (Q4) (Z = 2.4, p<0.02, uncorrected), and *react as though certain sounds were painful* (Q6) (Z = 1.7, p = 0.08, uncorrected), but did not differ on the other items (Q1, Q2, Q5, all p>0.16, uncorrected). The five children with highest ‘total auditory abnormality’ scores also had the highest ‘auditory sensitivity’ score, composed of items Q3, Q4, and Q6 (range 15–30 in these five ASD children vs 3–12 in the rest of the ASD group). The five children with marked auditory modulation difficulties did not significantly differ from the rest of the ASD sample in terms of age (high total score: 115 months, low total score: 134 months, t = −1.2, p = 0.24), general IQ (98 vs. 86, t = 1.3, p = 0.37) or AQ (83 vs. 89, t = −1.0, p = 0.34).

**Table 3:** Atypical auditory behavior during the 1^st^ two years of life: the mean and the range of the scores. Questions are adapted form Dahlgren & Gillberg (1998).

Question	ASD	TYP	*Z*
*Q1. He showed strange reactions to sound*	5.6 (1–10)	1.9 (1–7)	−2.5*
*Q2. A hearing deficit/deafness was suspected*	4.1(1–10)	1 (1–1)	−2.0
*Q3. He reacted strongly to sound, regardless of level*	4.8 (1–10)	2.1 (1–9)	−1.8
*Q4. He would often put his fingers in his ears*	3.1 (1–10)	1.2 (1–3)	−0.9
*Q5. He sometimes reacted strongly to barely audible sounds*	3.0 (1–10)	1.3 (1–5)	−1.4
*Q6. He reacted as though certain sounds were painful*	4.0 (1–10)	1.5 (1–8)	−1.7
*Total auditory abnormality score*	*24.5* (*6*–*58*)	*9.0* (*6*–*24*)	−3.5**

* 2-tailed Mann-Whitney U test. * p<0.05, **p<0.01, FDR corrected.

### P100m

**Figure 2:**
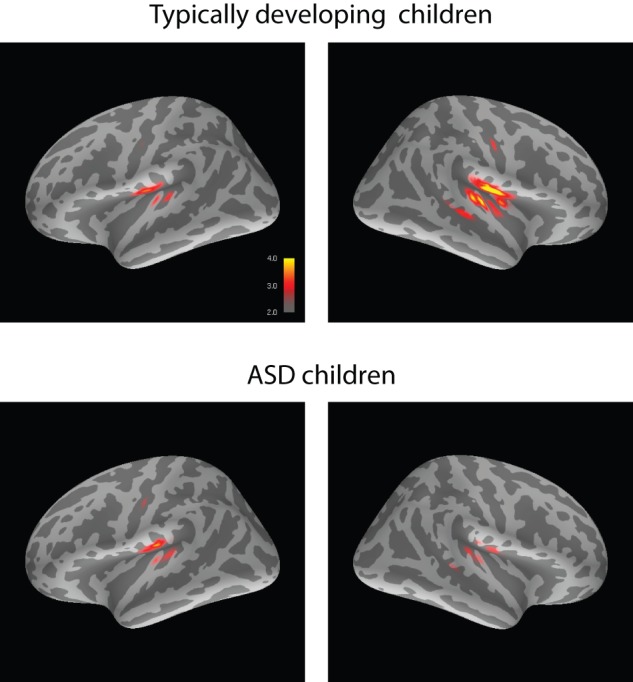
Source localization of the P100m in response to the first binaural click in a pair: group average absolute dSPM values at the component's peak.

Figure 2 displays source localization of the P100m based on the group-average of the absolute dSPM values for the first binaural click. Figure 1(B) shows the group average MNE time-courses in the left and right ROIs in the ASD and typically developing children.

Individual P100m peaks in response to both clicks (S1, S2) in both hemispheres were detected in 13 typically developing (mean age 127 months) and 12 ASD (mean age 131 months) subjects. A full-design ANOVA performed in these 25 subjects revealed no significant effects for the P100m latency (96 ms in control subjects and 99 ms in subjects with ASD).

**Figure 3:**
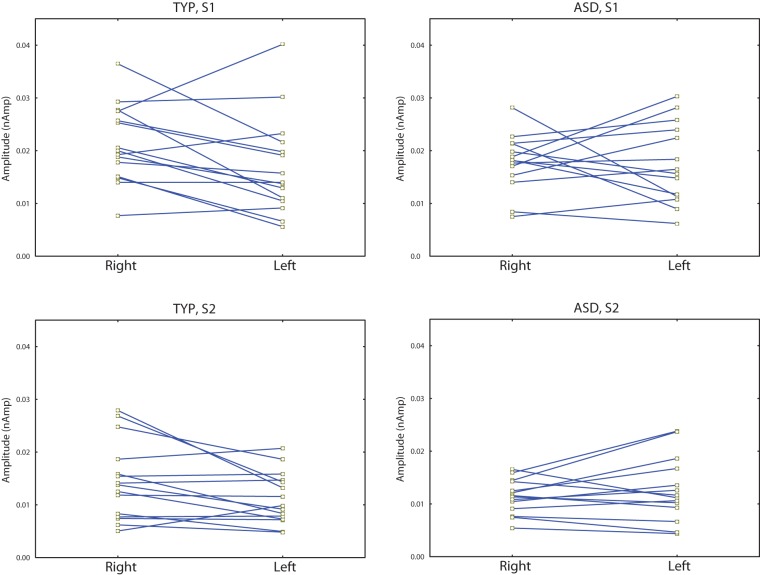
Individual P100m amplitude values in the typically developing (‘TYP’, 1^st^ column) and ASD (2^nd^ column) children in response to the 1^st^ (‘S1’, 1^st^ row) and second (‘S2’, 2^nd^ row) clicks in a pair.

**Figure 4:**
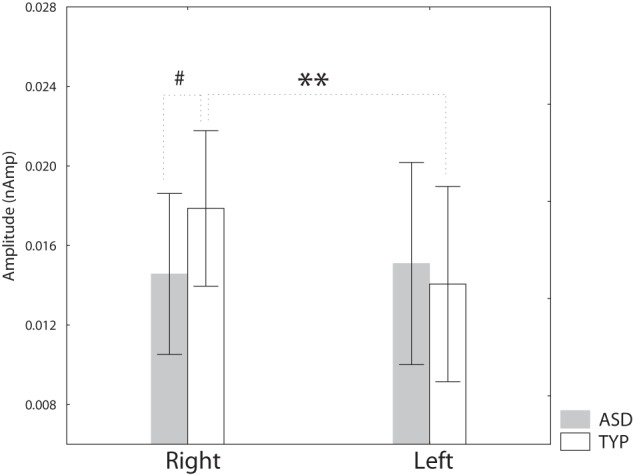
Group and hemispheric differences in the P100m amplitude in response to binaural clicks. *p<0.05, #p = 0.1. Vertical spreads denote 0.95 confidence intervals.

The individual amplitude scores in the two hemispheres are presented in figure 3. For P100m amplitude an ANOVA with factors Group, Stimulus Order and Hemisphere showed significant effect of the Stimulus Order (F_(1,27)_ = 46.7, p<0.0001), reflecting strong reduction of the P100m upon stimulus repetition. There was also significant Group*Hemisphere interaction (F_(1,27)_ = 4.9, p<0.05), which is visualized in figure 4. The typically developing children had higher P100m amplitude in the RH than in the LH (F_(1,27)_ = 7.9, p<0.01), while no hemispheric lateralization was found in the ASD group (F_(1,27)_ = 0.08, p = 0.8). The ASD children tended to have lower P100m amplitudes in the RH than the control children (F_(1,27)_ = 2.9, p = 0.1), while no group differences were found in the LH (F_(1,27)_ = 0.18, p = 0.7). In general this result suggests that the ASD children have atypical P100m lateralization in response to clicks.

### P100m hemispheric lateralization and behavior in the ASD children

Taking into account that no significant Group x Stimulus Order or Group x Stimulus Order x Hemisphere interactions were found, we further analyzed only P100m responses to the first binaural click, because of its higher amplitude and better signal-to-noise ratio. Table 4 shows correlation of P100m lateralization parameters with psychological variables and age in the ASD group. The P100m lateralization was calculated according to the formula: (RH−LH)/*(RH+LH).

**Table 4:** Correlations between P100m lateralization score and behavioral variables and age in the ASD children.

	Age	IQ	AQ	SSP-*total*
(*RH−LH*)*/*(*RH+LH*)	.05	−.43	.13	**.65** *

* p<0.05

The correlation of P100m inter-hemispheric asymmetry with SSP scores indicates less-rightward/more-leftward P100m lateralization in the ASD children with higher degree of sensory problems (Tab. 4). Correlations of the total SSP scores with either right or left P100m amplitude did not reach the significance level (Right: R = 0.46, p = 0.09; Left: R = −0.52, p = 0.053). Analysis of the separate SSP sections revealed significant correlation of the P100m asymmetry scores with Taste/Smell (R = 0.65, p<0.05, uncorrected), Movement Sensitivity (R = 0.54, p<0.05, uncorrected), and Low Energy/Weak (R = 0.56, p<0.05, uncorrected) sections.

No significant correlations between P100m lateralization scores and behavioral measures or age were found in the typically developing group.

**Figure 5:**
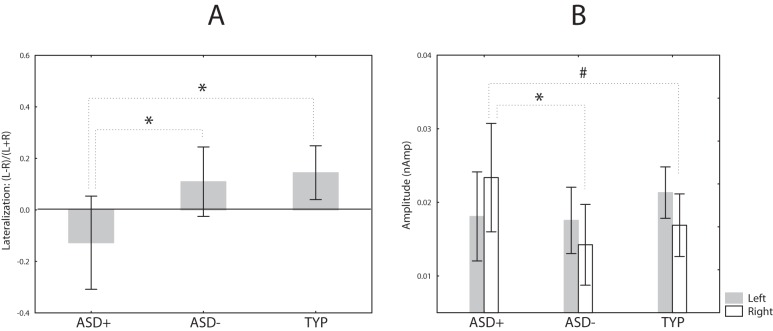
Comparison of the ASD children who experienced prominent auditory sensory modulation difficulties during the first two years of life (ASD+), with the ASD children with no or less prominent difficulties (ASD−), and typically developing control children. (A) The P100m lateralization. (B) Left and right P100m amplitude in response to the 1^st^ binaural click. *p<0.05, #p = 0.053, Mann-Whitney U test, 2-tailed. Vertical spreads denote 0.95 confidence intervals.

Next, we investigated relation between presence of considerable auditory modulation difficulties during the first two years of life and the P100m amplitude and lateralization in ASD children. We divided ASD children into two groups. The first group included five subjects who scored over maximal control group value on the total auditory abnormalities during first years of life (total scores were between 30 and 58). The rest of the ASD group (9 children) had no or less severe auditory modulation difficulties (total scores of 24 or below). Taking into account small and unequal sample sizes, we applied nonparametric Kruskal–Wallis one-way analysis of variance to test for the main effect of group. The main effect was significant (H _(2, N = 29)_  = 6.7 p<0.05) and we further applied the Mann-Whitney test to compare the ‘atypical auditory sensitivity group’ with the rest of the ASD sample, as well as with the typically developing children. The results of this comparison are plotted in figure 5 (A). ASD children with marked auditory modulation difficulties had more leftward-lateralized P100m than control children (Z = 2.5, p<0.05) or the ASD children without such difficulties (Z = 2.1, p<0.05). Further analysis has shown that the differences were mainly due to their higher P100m amplitude in the left hemisphere than in either control children (Z = −2.0, p = 0.05) or the ASD children without prominent auditory modulation difficulties (Z = −2.2, p<0.05), Fig. 5 (B).

## Discussion

This study demonstrates in ASD children atypical lateralization of the P100m component of the auditory field response to binaural clicks, and a tendency for its reduction in the RH. The atypical P100m lateralization correlated with presence of sensory modulation difficulties in ASD children. The small size of the ASD sample that included children with autism, PDD-NOS and Asperger syndrome, demands caution while interpreting these results. On the other hand, correlation of the P100m abnormalities with behavioral problems indicates that the atypical P100m lateralization may represent an important feature of the ASD phenotype.

### P100m abnormalities in the ASD children

The present study partly reproduced results of our previous EEG study in young children with and without autism [9]. Similarly to the previous EEG study, the present MEG study also revealed reduced lateralization of the cortical auditory response to binaural clicks and a tendency for the response reduction in the RH in ASD children.

The P100m response to binaural clicks was rightward lateralized in the typically developing children (Fig. 3, see also Fig. 2). We are not aware about other studies investigated hemispheric asymmetry of P100 or P100m to clicks in children. Therefore, the present results need replication by independent studies. At the same time, there is indirect evidence suggesting that the rightward hemispheric asymmetry of the auditory responses to certain types of auditory stimuli is a ‘normal’ finding. Specifically, the rightward lateralization in children has been previously found for the ‘radial’ component Tb in response to either clicks [9] or tones with sharp ramps [44]. The rightward lateralization of the midlatency component P50 to clicks has been reported in a few ‘sensory gating’ studies in adults [45]–[47]. The rightward amplitude and/or latency predominance of the N100(m) response to clicks and other auditory stimuli with sharp ramps has also been previously reported in adults in the EEG and MEG studies [14], [48], [49]. Importantly, the generally higher amplitude of N1 to clicks in the right vs left hemisphere have been observed even in intracranial records [50], suggesting functional rather than structural origin of this lateralization. The rightward lateralization effects may reflect greater RH involvement in such functions as stimulus-driven attention orienting, gating of conscious awareness of sounds, and processing of sound location [51]. The abnormal P100m lateralization in the ASD children with the tendency for decreased P100m amplitude in the RH may relate to abnormality of some of these normally right-lateralized functions.

The child P1 (P100m) is thought to reflect recurrent activation in primary and secondary auditory cortical areas [24]. Apart from specific auditory thalamic input the auditory cortical areas receive input from the nonspecific multisensory thalamic nuclei and medial pulvinar [52]–[54]. These nonspecific pathways are involved in arousal, spatial orienting and attention processes. It is likely that P100m is affected by these nonspecific influences. The strong habituation of this component upon stimulus repetition observed in our study provides indirect support for this view.

The role of modality nonspecific influences on generation of mid-latency positive components is also supported by pharmacological studies in human adults [28] and lesion studies in cats [55], which suggest that reticular activation strongly modulates the P1 component of human ERP or its animal analog ‘wave A’. Interestingly, Buchwald et al [27] reported decreased amplitude of the ‘vertex-positive’ P1 response to clicks in adults with high-functioning autism and proposed that the P1 reduction in autism reflects dysfunction of subcortical RAS system or of its cortical targets.

Taking into account the probable relation between P100m and RAS, the atypical P100m lateralization and the tendency for P100m reduction in the right hemisphere in ASD subjects may reflect abnormal preattentive arousal provoked by an abrupt sound. The fMRI studies suggest that, irrespective of stimulus modality, there seems to exist a mostly right-hemispheric cortical, thalamic, and brain-stem network which is coactivated by alerting and orienting attentional demands [12]. Conceivably, the hemispheric balance of the activation is disturbed in ASD at the preattentive stage of the auditory processing, mainly due to reduced activation in the right hemisphere.

What brain pathology underlies P100m abnormalities in ASD? Although the present study does not allow conclusions about origins of the observed P100m abnormalities, some speculations are possible.

Hypoperfusion in temporal lobes, including superior temporal cortex, has been found in approximately 75 percent (25/33) of children with autism [56]. Interestingly, analysis of individual results in the study of Zilbovicius et al (2000) revealed that the hypoperfusion was either bilateral (in 9 subjects) or located in the right hemisphere (in 16 subjects). The finding of predominantly rightward temporal hypoperfusion in autism may be related to the right-hemispheric P100m reduction in ASD in the present study.

The role of subcortical structures, such as brainstem and thalamus is also plausible. A contribution of brainstem pathology to both sensory and social symptoms of autism has been proposed long ago by Ornitz [57]. Correlations between reduced brain stem gray matter volume and sensory modulation difficulties has recently been found in children with autism [58]. The other candidate structure is the thalamus. Thalamus is the main relay hub between subcortical structures and the cerebral cortex that conveys specific sensory modality information as well as ‘nonspecific’ influences from the reticular formation. Multiple studies have reported on thalamic abnormalities in autism and ASD [59]–[67]. Hardan and colleagues reported some correlations between altered thalamic metabolism and sensory modulation difficulties in children with autism [62]. Interestingly, nicotinic abnormalities were found in adults with autism that were limited to ‘nonspecific’ midline thalamic nuclei innervated by the reticular formation [68]. A recent diffusion tensor imaging study [69] found decreased fractional anisotropy in the anterior thalamic radiation (ATR) in high-functioning boys with ASD. ART connect ‘nonspecific’ mediodorsal and anterior thalamic nuclei with the frontal and the anterior cingulate cortices. Noteworthy, greater severity of autism symptoms in the study of Cheon et al correlated with decreased fractional anisotropy in ATR of the RH, suggesting a role for ‘nonspecific’ right thalamo-cortical projections in autism symptomatology.

### The role of ISIs

Unlike our previous study [9], the present study did not provide evidence for a role of temporal novelty (long ISIs) in the normal rightward lateralization of the auditory response, or in its RH reduction in children with autism. There may be at least two reasons for this discrepancy. *First*, these ISI effects may be specific for the radial N1c (Tb) sources situated at the lateral surface of the temporal lobe [15] and not detected in our MEG study. *Second*, a phase cancellation of the P100(m) by the emerging N100(m) in the older children in the present study could be a confounding factor [15], [23]. Both lateralization of P100(m) and its putative modulation by ISIs could possibly be better detected in younger children who have not yet started developing the N100(m) component of the opposite polarity.

### P100m and sensory abnormalities in the ASD children

The ASD children in our study had sensory modulation difficulties in different sensory domains (Tab. 2), that agree with results of many behavioral studies in ASD [1]–[3], [31]–[33]. The ASD children also had sensory modulation difficulties in the auditory domain during early life (Tab. 3).

The lack of normal rightward lateralization of P100m in ASD children was related to greater severity of sensory modulation abnormalities across different sensory domains, as indicated by correlation of the P100m hemispheric asymmetry with the total SSP score (Tab. 4). It was also related to the presence of early auditory sensory modulation difficulties (Fig. 4 B). It has been proposed that the RH is dominant for rapid, global and rough detection of stimuli, as well as rapid transfer of the information to the LH for more detailed processing [11]. It is conceivable that dysfunction of these processes in the RH and greater reliance on a ‘non-optimal’ LH during early processing of some stimulation features may contribute to sensory abnormalities observed in ASD.

Interestingly, the five ASD children who had marked auditory modulation difficulties during early life had higher P100m amplitude in the LH than either ASD children with no/less prominent difficulties or typically developing children, while no such differences were found in their RH (Fig. 5 B). It seems that heightened sensitivity to sound observed in these children was reflected in relatively increased processing in the LH only.

### Limitations

There are a few limitation of the present study. The obvious one is the small number of subjects. Reproduction of the finding in a greater sample is needed to draw firm conclusions about behavioral correlates of the P100m abnormalities in ASD children. Future studies should also consider investigation of more homogeneous age groups. Investigation of younger children (before 9 years of age) would preclude possible cancellation of the P100m by a developing N100m wave and could appear especially informative. Investigation of other control groups, such as children with sensory modulation difficulties without autism, would help to assess specificity of the present findings for ASD individuals. Last, we would like to stress that we did not aim to localize sources of the observed P100m effects with high precision, since the MNE/dSPM method has known localization bias [70].

### Conclusions

In conclusion, our results suggest that amplitude of the P100m response to clicks, and abnormal P100m hemispheric lateralization, are related to sensory behavioral abnormalities in the ASD children. We propose that the P100m component in responses to clicks may provide a valuable indicator of pre-attentive arousal disturbances in ASD children, especially in young children, in whom P100m is the dominating early component of the auditory response.

## Supporting Information

Material S1 **Response to the first binaural click in one typically developing 10-years-old boy.** (A) dSPM values (with sign) at the peaks of the P100m and N100m components. The dSPM values greater than 4.03 or lower than −4.03 are significant at p<0.01 (two-tailed F-test). Different scales were used for P100m and N100m. Red to yellow and blue to light-blue colors correspond to outgoing vs. ingoing currents. Note reversion off current direction between 92 and 134 ms at the superior temporal area (outlined in white). (B) Modeling with a single dipole source, saggital view. Note the top/frontal direction of the P100m and backwards/down direction of the N100m dipole sources. Note that positions and orientations of the dipole sources modeling P100m and N100m currents are similar to those described for P50m (P1m) and N100m in adults (Hanlon et al., 2005). (C) The dSPM time course of one vertex source at the Herschl gyrus.
(DOC)

Material S2 **The superior temporal regions used to measure P100m latency**. These regions were defined as aggregates of superior temporal sources displaying positive activation in the P100m time range in more than 50% of the subjects. The source was considered activated if it demonstrated significant (p<0.05) positive dSPM value at some time point within 76–130 ms interval. The mean time courses of MNE current were calculated in these regions and P100m latencies were measured at the left and right peaks in 76–130 ms range.
(DOC)

## References

[1] Ben-Sasson A, Cermak SA, Orsmond GI, Tager-Flusberg H, Kadlec MB (2008). Sensory clusters of toddlers with autism spectrum disorders: differences in affective symptoms.. Journal of Child Psychology and Psychiatry.

[2] Leekam SR, Nieto C, Libby SJ, Wing L, Gould J (2007). Describing the sensory abnormalities of children and adults with autism.. Journal of Autism and Developmental Disorders.

[3] Liss M, Saulnier C, Kinsbourne DF, Kinsbourne M (2006). Sensory and attention abnormalities in autistic spectrum disorders.. Autism.

[4] Crane L, Goddard L, Pring L (2009). Sensory processing in adults with autism spectrum disorders.. Autism.

[5] Harrison J, Hare DJ (2004). Brief report: Assessment of sensory abnormalities in people with autistic spectrum disorders.. Journal of Autism and Developmental Disorders.

[6] Dahlgren SO, Gillberg C (1989). Symptoms in the 1st 2 years of life – a preliminary population study of infantile-autism.. European Archives of Psychiatry and Clinical Neuroscience.

[7] Stiegler LN, Davis R (2010). Understanding Sound Sensitivity in Individuals with Autism Spectrum Disorders.. Focus on Autism and Other Developmental Disabilities.

[8] van Engeland H (1984). The electrodermal orienting response to auditive stimuli in autistic children, normal children, mentally retarded children, and child psychiatric patients.. J Autism Dev Disord.

[9] Orekhova EV, Stroganova TA, Prokofiev A, Nygren G, Gillberg C (2009). The right hemisphere fails to respond to temporal novelty in autism: Evidence from an ERP study.. Clinical Neurophysiology.

[10] Corbetta M, Patel G, Shulman GL (2008). The reorienting system of the human brain: From environment to theory of mind.. Neuron.

[11] Okon-Singer N, Podlipsky I, Siman-Tov T, Ben-Simon E, Zhdanov A (2011). Spatio-temporal indications of sub-cortical involvement in leftward bias of spatial attention.. Neuroimage.

[12] Sturm W, Willmes K (2001). On the functional neuroanatomy of intrinsic and phasic alertness.. Neuroimage.

[13] Brugge JF, Volkov IO, Oya H, Kawasaki H, Reale RA (2008). Functional localization of auditory cortical fields of human: Click-train stimulation.. Hearing Research.

[14] Howard MA, Volkov IO, Mirsky R, Garell PC, Noh MD (2000). Auditory cortex on the human posterior superior temporal gyrus.. Journal of Comparative Neurology.

[15] Ponton C, Eggermont JJ, Khosla D, Kwong B, Don M (2002). Maturation of human central auditory system activity: separating auditory evoked potentials by dipole source modeling.. Clinical Neurophysiology.

[16] Cardy JEO, Ferrari P, Flagg EJ, Roberts W, Roberts TPL (2004). Prominence of M50 auditory evoked response over M100 in childhood and autism.. Neuroreport.

[17] Cardy JEO, Flagg EJ, Roberts W, Roberts TPL (2008). Auditory evoked fields predict language ability and impairment in children.. International Journal of Psychophysiology.

[18] Fujioka T, Ross B, Kakigi R, Pantev C, Trainor LJ (2006). One year of musical training affects development of auditory cortical-evoked fields in young children.. Brain.

[19] Paetau R, Ahonen A, Salonen O, Sams M (1995). Auditory-evoked magnetic-fields to tones and pseudowords in healthy-children and adults.. Journal of Clinical Neurophysiology.

[20] Ruhnau P, Herrmann B, Maess B, Schroger E (2011). Maturation of obligatory auditory responses and their neural sources: Evidence from EEG and MEG.. Neuroimage.

[21] Kotecha R, Pardos M, Wang YY, Wu T, Horn P (2009). Modeling the Developmental Patterns of Auditory Evoked Magnetic Fields in Children.. Plos One 4, e4811.

[22] Sussman E, Stemschneider M, Gumenyuk V, Grushko J, Lawson K (2008). The maturation of human evoked brain potentials to sounds presented at different stimulus rates.. Hearing Research.

[23] Eggermont JJ, Ponton CW (2003). Auditory-evoked potential studies of cortical maturation in normal hearing and implanted children: Correlations with changes in structure and speech perception.. Acta Oto-Laryngologica.

[24] Kral A, Eggermont JJ (2007). What's to lose and what's to learn: Development under auditory deprivation, cochlear implants and limits of cortical plasticity.. Brain Research Reviews.

[25] Moore JK, Linthicum FH (2007). The human auditory system: A timeline of development.. International Journal of Audiology.

[26] Moore JK, Guan YL (2001). Cytoarchitectural and axonal maturation in human auditory cortex.. Jaro.

[27] Buchwald JS, Erwin R, Vanlancker D, Guthrie D, Schwafel J (1992). Midlatency auditory evoked-responses – p1 abnormalities in adult autistic subjects.. Electroencephalography and Clinical Neurophysiology.

[28] Buchwald JS, Rubinstein EH, Schwafel J, Strandburg RJ (1991). Midlatency auditory evoked-responses – differential-effects of a cholinergic agonist and antagonist.. Electroencephalography and Clinical Neurophysiology.

[29] Dunn W (1999). Sensory profile: user's manual. San Antonio: Psychological Corp.. xiv, 146 p.

[30] Rogers SJ, Hepburn S, Wehner E (2003). Parent reports of sensory symptoms in toddlers with autism and those with other developmental disorders.. Journal of Autism and Developmental Disorders.

[31] Tomchek SD, Dunn W (2007). Sensory processing in children with and without autism: A comparative study using the short sensory profile.. American Journal of Occupational Therapy.

[32] Wiggins LD, Robins DL, Bakeman R, Adamson LB (2009). Breif Report: Sensory Abnormalities as Distinguishing Symptoms of Autism Spectrum Disorders in Young Children.. Journal of Autism and Developmental Disorders.

[33] Kern JK, Trivedi MH, Garver CR, Grannemann BD, Andrews AA (2006). The pattern of sensory processing abnormalities in autism.. Autism.

[34] Berument SK, Rutter M, Lord C, Pickles A, Bailey A (1999). Autism screening questionnaire: diagnostic validity.. British Journal of Psychiatry.

[35] Auyeung B, Baron-Cohen S, Wheelwright S, Allison C (2008). The Autism Spectrum Quotient: Children's Version (AQ-Child).. Journal of Autism and Developmental Disorders.

[36] Delorme A, Makeig S (2004). EEGLAB: an open source toolbox for analysis of single-trial EEG dynamics including independent component analysis.. Journal of Neuroscience Methods.

[37] Dale AM, Fischl B, Sereno MI (1999). Cortical surface-based analysis – I. Segmentation and surface reconstruction.. Neuroimage.

[38] Fischl B, Sereno MI, Dale AM (1999). Cortical surface-based analysis – II: Inflation, flattening, and a surface-based coordinate system.. Neuroimage.

[39] Hamalainen MS, Ilmoniemi RJ (1994). Interpreting magnetic-fields of the brain: minimum norm estimates.. Medical & Biological Engineering & Computing.

[40] Lin FH, Witzel T, Ahlfors SP, Stufflebeam SM, Belliveau JW (2006). Assessing and improving the spatial accuracy in MEG source localization by depth-weighted minimum-norm estimates.. Neuroimage.

[41] Lin FH, Belliveau JW, Dale AM, Hamalainen MS (2006). Distributed current estimates using cortical orientation constraints.. Human Brain Mapping.

[42] Dale AM, Liu AK, Fischl BR, Buckner RL, Belliveau JW (2000). Dynamic statistical parametric mapping: Combining fMRI and MEG for high-resolution imaging of cortical activity.. Neuron.

[43] Benjamini Y, Yekutieli D (2001). The control of the false discovery rate in multiple testing under dependency.. Annals of Statistics.

[44] Bishop DVM, Anderson M, Reid C, Fox AM (2011). Auditory Development between 7 and 11 Years: An Event-Related Potential (ERP) Study.. Plos One 6.

[45] Huang MX, Edgar JC, Thoma RJ, Hanlon FM, Moses SN (2003). Predicting EEG responses using MEG sources in superior temporal gyrus reveals source asynchrony in patients with schizophrenia.. Clinical Neurophysiology.

[46] Thoma RJ, Hanlon FM, Moses SN, Edgar JC, Huang MX (2003). Lateralization of auditory sensory gating and neuropsychological dysfunction in schizophrenia.. American Journal of Psychiatry.

[47] Weiland BJ, Boutros NN, Moran JM, Tepley N, Bowyer SM (2008). Evidence for a frontal cortex role in both auditory and somatosensory habituation: A MEG study.. Neuroimage.

[48] Blumenfeld LD, Clementz BA (2001). Response to the first stimulus determines reduced auditory evoked response suppression in schizophrenia: single trials analysis using MEG.. Clinical Neurophysiology.

[49] Hine J, Debener S (2007). Late auditory evoked potentials asymmetry revisited.. Clinical Neurophysiology.

[50] Boutros NN, Gjini K, Urbach H, Pflieger ME (2011). Mapping Repetition Suppression of the N100 Evoked Response to the Human Cerebral Cortex.. Biological Psychiatry.

[51] Howard MF, Poeppel D (2009). Hemispheric asymmetry in mid and long latency neuromagnetic responses to single clicks.. Hearing Research.

[52] Budinger E, Heil P, Hess A, Scheich H (2006). Multisensory processing via early cortical stages: Connections of the primary auditory cortical field with other sensory systems.. Neuroscience.

[53] Hackett TA, Stepniewska I, Kaas JH (1998). Thalamocortical connections of the parabelt auditory cortex in macaque monkeys.. Journal of Comparative Neurology.

[54] Romanski LM, Giguere M, Bates JF, GoldmanRakic PS (1997). Topographic organization of medial pulvinar connections with the prefrontal cortex in the rhesus monkey.. Journal of Comparative Neurology.

[55] Harrison JB, Woolf NJ, Buchwald JS (1990). Cholinergic neurons of the feline pontomesencephalon.1. essential role in wave-a generation.. Brain Research.

[56] Zilbovicius M, Boddaert N, Belin P, Poline JB, Remy P (2000). Temporal lobe dysfunction in childhood autism: A PET study.. American Journal of Psychiatry.

[57] Ornitz EM (1983). The functional neuroanatomy of infantile-autism.. International Journal of Neuroscience.

[58] Jou RJ, Minshew NJ, Melhem NM, Keshavan MS, Hardan AY (2009). Brainstem volumetric alterations in children with autism.. Psychological Medicine.

[59] Friedman SD, Shaw DW, Artru AA, Richards TL, Gardner J (2003). Regional brain chemical alterations in young children with autism spectrum disorder.. Neurology.

[60] Hardan AY, Girgis RR, Adams J, Gilbert AR, Keshavan MS (2006). Abnormal brain size effect on the thalamus in autism.. Psychiatry Research-Neuroimaging.

[61] Hardan AY, Girgis RR, Adams J, Gilbert AR, Melhem NM (2008). Brief report: Abnormal association between the thalamus and brain size in Asperger's disorder.. Journal of Autism and Developmental Disorders.

[62] Hardan AY, Minshew NJ, Melhem NM, Srihari S, Jo B (2008). An MRI and proton spectroscopy study of the thalamus in children with autism.. Psychiatry Research-Neuroimaging.

[63] Haznedar MM, Buchsbaum MS, LiCalzi EM, Cartwright C, Hollander E (2006). Volumetric analysis and three-dimensional glucose metabolic mapping of the striatum and thalamus in patients with autism spectrum disorders.. American Journal of Psychiatry.

[64] Spencer MD, Moorhead TWJ, Lymer GKS, Job DE, Muir WJ (2006). Structural correlates of intellectual impairment and autistic features in adolescents.. Neuroimage.

[65] Tamura R, Kitamura H, Endo T, Hasegawa N, Someya T (2010). Reduced thalamic volume observed across different subgroups of autism spectrum disorders.. Psychiatry Research-Neuroimaging.

[66] Tsatsanis KD, Rourke BP, Klin A, Volkmar FR, Cicchetti D (2003). Reduced thalamic volume in high-functioning individuals with autism.. Biological Psychiatry.

[67] Waiter GD, Williams JHG, Murray AD, Gilchrist A, Perrett DI (2004). A voxel-based investigation of brain structure in male adolescents with autistic spectrum disorder.. Neuroimage.

[68] Ray MA, Graham AJ, Lee M, Perry RH, Court JA (2005). Neuronal nicotinic acetylcholine receptor subunits in autism: An immunohistochemical investigation in the thalamus.. Neurobiology of Disease.

[69] Cheon KA, Kim YS, Oh SH, Park SY, Yoon HW (2011). Involvement of the anterior thalamic radiation in boys with high functioning autism spectrum disorders: A Diffusion Tensor Imaging study.. Brain Research.

[70] Hauk O, Wakeman DG, Henson R (2011). Comparison of noise-normalized minimum norm estimates for MEG analysis using multiple resolution metrics.. Neuroimage.

