# Supplementary material for: Auditory Cortex Responses to Clicks and Sensory Modulation Difficulties in Children with Autism Spectrum Disorders (ASD)
Source: PLoS One. 2012 Jun 29;7(6):e39906. doi: 10.1371/journal.pone.0039906 (PMC3387220; doi:10.1371/journal.pone.0039906)
Supplement: Material S1 — Response to the first binaural click in one typically developing 10-years-old boy. (A) dSPM values (with sign) at the peaks of the P100m and N100m components. The dSPM values greater than 4.03 or lower than −4.03 are significant at p<0.01 (two-tailed F-test). Different scales were used for P100m and N100m. Red to yellow and blue to light-blue colors correspond to outgoing vs. ingoing currents. Note reversion off current direction between 92 and 134 ms at the superior temporal area (outlined in white). (B) Modeling with a single dipole source, saggital view. Note the top/frontal direction of the P100m and backwards/down direction of the N100m dipole sources. Note that positions and orientations of the dipole sources modeling P100m and N100m currents are similar to those described for P50m (P1m) and N100m in adults (Hanlon et al., 2005). (C) The dSPM time course of one vertex source at the Herschl gyrus. (DOC) [file pone.0039906.s001.doc]

**Supplementary material 1**

The P100m and N100m in children are close in time and it is sometimes difficult to separate between these components based on the absolute current values. The direction of current, however, clearly separates between these components (see the figure).
