# Supplementary material for: Auditory Cortex Responses to Clicks and Sensory Modulation Difficulties in Children with Autism Spectrum Disorders (ASD)
Source: PLoS One. 2012 Jun 29;7(6):e39906. doi: 10.1371/journal.pone.0039906 (PMC3387220; doi:10.1371/journal.pone.0039906)
Supplement: Material S2 — The superior temporal regions used to measure P100m latency. These regions were defined as aggregates of superior temporal sources displaying positive activation in the P100m time range in more than 50% of the subjects. The source was considered activated if it demonstrated significant (p<0.05) positive dSPM value at some time point within 76–130 ms interval. The mean time courses of MNE current were calculated in these regions and P100m latencies were measured at the left and right peaks in 76–130 ms range. (DOC) [file pone.0039906.s002.doc]

**Supplementary material 2**
